# Obesity in French Inmates: Gender Differences and Relationship with Mood, Eating Behavior and Physical Activity

**DOI:** 10.1371/journal.pone.0170413

**Published:** 2017-01-19

**Authors:** Aude Lagarrigue, Soufiane Ajana, Lucile Capuron, Catherine Féart, Marie-Pierre Moisan

**Affiliations:** 1 CHU Rangeuil, Service de médecine légale et médecine en milieu pénitentiaire, Toulouse, France; 2 INSERM, ISPED, Centre INSERM U1219-Bordeaux Population Health, Bordeaux, France; 3 Univ. Bordeaux, ISPED, Centre INSERM U1219- Bordeaux Population Health, Bordeaux, France; 4 INRA, Nutrition et neurobiologie intégrée, UMR 1286, Bordeaux, France; 5 Univ. Bordeaux, Nutrition et neurobiologie intégrée, UMR 1286, Bordeaux, France; University of Oslo, NORWAY

## Abstract

**Context:**

Inmates, notably women, are at greater risk for obesity and metabolic complications than the general population according to several studies from high income countries. Data regarding French correctional institutions are lacking so far. To fill this gap, we have assessed in a sample from a French prison (33 females and 18 males) the gender-specific effect of incarceration on weight and body mass index (BMI) and examined their current metabolic status. Furthermore, to reveal the possible determinants of increased obesity, we analyzed emotional vulnerability, eating behavior and physical activity using self-reported questionnaires.

**Results:**

In this sample, obesity (BMI≥30 kg/m^2^) was already frequent in women (18.2%) but rather scarce for men (11%) at prison entry. Incarceration worsened the rate of obesity in both genders (21.2% and 16.7% respectively). At the time of study, abdominal obesity estimated through waist circumference was particularly prevalent in women (69.7%) versus men (27.8%) and metabolic syndrome was detected in 33% of female against none in male inmates. Abdominal obesity was associated with female sex (p<0.03), low physical activity (p<0.05) and eating disorder (p = 0.07) in univariate analyses. Low physical activity remained significant as an explanatory factor of higher abdominal obesity in multivariate analysis. A marked difference between genders was found for practice of physical activity with a higher proportion of women compared to men being inactive (37.9% vs. 11.8%) and fewer women being very active (17.2% vs. 41.2%).

**Conclusion:**

This study revealed that a significant proportion of women of this correctional institution combined established obesity, a metabolic syndrome and very little practice of physical activity which put them at high risk of cardiovascular disease. Thus, obesity should be better surveyed and treated in prison, especially for female inmates. Increased physical activity, adapted to obese women, would be the first mean to decrease obesity and gender differences.

## Introduction

Among the most prevalent non-communicable diseases, obesity and diabetes are reported as major problems for the general population and represent thus important public health issues. Unhealthy diets and inadequate physical activity are the main factors contributing to overweight, obesity and type 2 diabetes [1]. Some populations are particularly vulnerable to these disorders. For instance, inmates are often mentioned, especially women. A recent systematic review including 13 studies with a total of 46711 prisoners (39631 [85%] men and 7080 [15%] women) reported that male prisoners were less likely to be obese than males in the general population (prevalence ratio ranged from 0.33 to 0.87, except one study in USA 1.02) whereas female prisoners were more likely to be obese than non-imprisoned women (prevalence ratio 1.15 to 1.18 except one study in UK 0.70) [2].

So far, there is no study interested on the relationship between incarceration and overweight, obesity or weight change in French correctional institutions. Consequently, the effect of gender on this relationship is still unknown. However, in the general French population, obesity rate is higher in women than in men and increases more rapidly in women (from 8.3% in 1997 to 15.7% in 2012) than in men (8.8% in 1997 to 14.3% in 2012) [3]. For both sexes, obesity rate is inversely proportional to income level, reaching 24.1% in the poorest part of the population. Obesity increases gradually with age, peaking at 19.5% between age 55–64 with no more gender difference after 55 year of age [3]. Given that most prisoners come from the poorest and most marginalized sections of the society and since the incarcerated population is aging, prisoners may be at greater risk for obesity and its complications, especially women. To test this hypothesis, we conducted a pilot study in a French correctional institution to assess the effect of incarceration on weight and obesity status and to investigate the influence of gender. Furthermore, to study the possible determinants of increased weight and obesity parameters we analyzed emotional vulnerability, physical activity and eating behavior in the same enrolled participants. We found that indeed a large proportion of incarcerated women of this French prison are obese with metabolic complications. Lack of physical activity appears as a major explanatory factor.

## Method

### Participants

The study was conducted from September 2012 to September 2013 in inmates from the adult correctional institution of Seysse’s, France, a facility that holds individuals before trial or sentencing, or individuals serving short sentences (less than 1 year). There were on average, during the study period, 46 female inmates in this prison, who were all informed of the study by mail and during a medical visit conducted by the physician investigator (AL). The study was then proposed to a sub-sample of male inmates that match the recruited women in terms of age, length of incarceration and judiciary status (detainee vs. sentenced). Pregnant or breast-feeding female subjects were excluded from the study. Written informed consent was obtained from each participant. The study was approved by the local research ethic committee of Rangeuil hospital, Toulouse, France in July 2012 (project # 38–0712).

### Study design

All participants were given the questionnaires related to mood, coping strategies, traumatic history, eating behaviors and physical activity with the instruction to fulfill the documents alone. Later on, they were called for a specific medical visit where the physician investigator collected the filled questionnaires, as well as demographic (age, marital status, number of children, educational attainment, previous occupation), judiciary (length of incarceration, detainee vs. sentenced status, presence of subsequent offence, work in prison, indigence, number of visit per month) and clinical/medical information (smoking, drug addiction, alcoholism, presence and type of specific diet, previous medical history, medication before and since incarceration). During this visit, the weight, waist circumference (WC) and arterial pressure of study participants were measured and a blood sample was drawn by a nurse for further biological assessments (i.e. presence of metabolic syndrome). For the weight, the participants were without clothes and fasted. The height was checked with a height wall gauge. WC was measured as the minimum value between the iliac crest and the lateral costal margin using a meter tape. Blood pressure was measured once, after a rest. Participants were fasted overnight before the blood sampling. The height and weight of each participant when entering prison were measured at prison entry and available to the physician from the prison’s medical records.

### Assessments

#### Weight and metabolic parameters

Body mass index (BMI) when entering prison and BMI at the time of the study (i.e., during incarceration) were calculated from measured height and weight and individuals were categorized as underweight (<18.5 kg/m^2^), normal weight (18.5 kg/m^2^≤BMI<25 kg/m^2^), overweight (25 kg/m^2^≤BMI<30 kg/m^2^), or obese (BMI ≥30 kg/m^2^). Abdominal obesity was defined by a WC ≥ 80 cm for women and WC ≥ 94 cm for men, as per International Diabetes Federation (IDF) [4]. Weight change was calculated from weight measured at prison admittance and current weight at the time of the study. Fasting glycemia, glycated hemoglobin (HbA1C), triglycerides (TG), LDL- and HDL- cholesterol were measured from the subjects’ blood samples by routine electrophoresis (HbA1C) or enzymatic (Glycemia, TG, HDL/LDL) analyses. Metabolic syndrome was defined according to the criteria provided by the IDF [4], i.e. abdominal obesity plus any two of the following criteria: raised TG≥1.7 mmol/l, reduced HDL cholesterol <1.29 mmol/l in females, <1.03 mmol/l in males, raised systolic blood pressure ≥130 mmHg or diastolic ≥85 mmHg, raised fasting glucose ≥5.6 mmol/l. Presence of atherogenesis was estimated by an atherogenic index (ratio of total cholesterol/HDL cholesterol) over 4.5.

#### Mood symptoms and emotional vulnerability

Psychometric self-report questionnaires were used to assess depressive symptoms, anxiety, traumatic history and coping strategies. The intensity of depressive symptomatology was evaluated with the French version of the 13-item Beck Depression Inventory (BDI) [5]. BDI scores can be categorized as follows: 0–3 = no, 4–7 = light, 8–15 = moderate ≥16 = severe depressive symptoms. Trait and state anxiety were assessed using the Spielberger State-Trait Anxiety Inventory Y form (STAI-Y) [6]. The scale STAI Y-A evaluates “state anxiety” defined as a transitory emotional state whereas the scale STAI Y-B evaluates “trait anxiety” defined as a personality trait considered as being a relatively stable tendency “to perceive stressful situations as dangerous or threatening, and to respond to such situations with elevations in the intensity of state anxiety reactions”. The following median norms were used to classify the participants: STAI-B = 40 in women, STAI-B = 35 in men [7]. History of trauma was determined using the traumatic history questionnaire (THQ), a history collection instrument that explores various types of trauma (general traumatic life events, crimes, physical/sexual trauma) [8], and coping strategies were measured with the French version of the 29-item Ways of Coping Checklist [9]. Norms for each dimension of these questionnaires were defined as early reported (8, 19).

#### Eating behaviors

The French version of the Questionnaire on Eating and Weight Patterns (QEWP) was used as a continuous score with a score above 8 indicating binge eating disorder, as earlier proposed in a non patient community sample [10]. The Dutch Eating Behavior Questionnaire (DEBQ) was used to evaluate 3 distinct factors of eating behavior: food restriction operated by the subject (restrained eating), emotional eating and externality (sensitivity to external cues), each expressed as continuous scores [11]. The norms for this test in females/males were for restrained eating: 2.49/1.84, for emotional eating: 2.06/1.72, for external eating: 2.68/2.64.

#### Physical activity

Physical activity was assessed with the International Physical Activity Questionnaire (IPAQ) short version. Scoring was performed following recommendations published on www.ipaq.ki.se (guidelines for data processing and analysis). Continuous scores were expressed as MET level x minutes of activity x events per week where MET = metabolic equivalent task, a physiological measure expressing the energy cost of physical activities. The 3 categories, inactive, minimally active and very active (or HEPA for health enhancing physical activity) where determined as recommended (www.ipaq.ki.se).

### Statistical analyses

Sociodemographic, judiciary, clinical and physical characteristics were described by their medians, interquartile range (IQR) and distributions. We firstly compared these characteristics based on gender, and then between participants without (i.e. control) and with abdominal adiposity (i.e. those with a WC over the sex-specific thresholds) using the Mann Whitney Wilcoxon's test (due to the sample size) and Chi-Square or Fisher's exact tests when appropriate. Spearman correlations were performed between the various psychological assessments.

The sample size being too small for a regular logistic regression, multivariate exact logistic regression was used to identify the socio-demographic, judiciary, clinical and physical variables that were independently associated with prevalence of abdominal adiposity. The variables included in this analysis were those associated with abdominal adiposity at *p*≤0.05 in crude univariate analysis, except sex to avoid over-adjustment (i.e. WC is defined by sex-specific thresholds). We chose this threshold (*p*≤0.05) because we did not have enough power (only 51 observations available) to run an exact logistic regression on a higher number of covariates. IPAQ categories and QEWP scores were both maintained as the 2 main explanatory variables in the regression model. IPAQ categories were preferred to IPAQ MET continuous score because of the highly asymmetric distribution of this continuous score. The SAS statistical package (Version 9.3 SAS Institute) was used for these analyses.

## Results

### Characteristics of participants and gender differences

#### Demographic and judiciary data

Characteristics of study participants are shown in Table 1. Among the 46 women imprisoned at the time of the study, 3 were pregnant, 1 was retained in disciplinary district and 9 refused to participate, resulting in 33 female volunteers. Only 18 men, matched to women for age, judiciary status and duration of incarceration, were enrolled, mainly due to a lack of interest for the study. The median age of the cohort was 41 y (IQR 14 y), 54.9% of inmates were sentenced and the median duration of incarceration was 10 months (IQR 26 months) with no statistical difference between genders as ensured by the study design. There were no statistical gender differences for education level, familial status or number of children. A large proportion of female (69.7%) and of male (50.0%) inmates was regular smokers. The same percentage of female and male inmates worked in prison (61%). First incarceration and the number of visit per month from family or friends were more frequent in women but not statistically different from men.

**Table 1:** Characteristics of study participants regarding demographic and judiciary data.

			Women (n = 33)	Men (n = 18)	P value
**Demographic data**					
	Age, median (IQR)		40 (14)	43 (16)	0.20
	Education	%, (n)			0.18
		None	6.0 (2)	0 (0)	
		primary	9.0 (3)	11. 1 (2)	
		secondary	57.6 (19)	83.3 (15)	
		over	27.3 (9)	16.7 (1)	
	Familial status, %, (n)				0.24
		single	45.4 (15)	38.9 (7)	
		married/couple	36.4 (12)	22.2 (4)	
		divorced/separated	18. 2 (6)	38.9 (7)	
	Number of children				
		Median (IQR)	2.0 (4)	1.0 (2)	0.29
	Smoking status	%, (n)	69.7 (23)	50.0 (9)	0.17
**Judiciary data**					
	Penal status				
		% sentenced, (n)	57.6 (19)		50.0 (9)	0.60
	Length of incarceration (months)				
		Median (IQR)	7.0 (21)	10.5 (25)	0.50
	First incarceration %, (n)		42.4 (14)	27.8 (5)	0.30
	Number of visit/month				
		Median (IQR)	2.0 (4)	1.5 (4)	0.50
	Work during incarceration %, (n)		60.6 (20)	61.1 (11)	0.97

#### Weight parameters and metabolic syndrome

**Fig 1:**
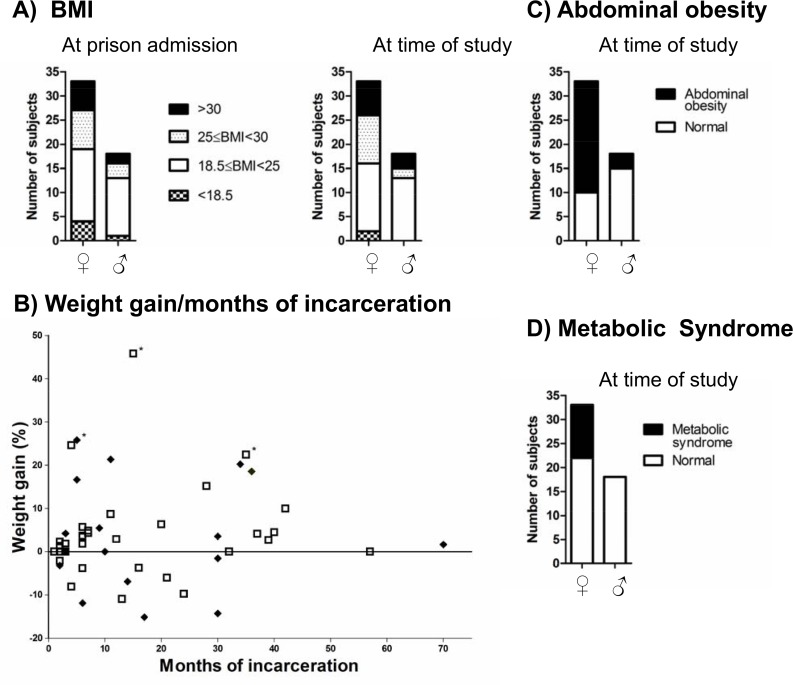
Body Mass Index (BMI) in kg/m^2^ (A), weight change (B), abdominal obesity (C), and metabolic syndrome (D) in participants. White squares = women, black diamond = men, * individual underweight on prison reception.

At the time of prison entry, 24.2% of females and 16.7% of males were overweight, and 18.2% of females and 11.1% of males were obese (Fig 1A). Between prison entry and the time of the study, only a small majority of inmates of both sexes gained weight (57.6% of women and 55.5% of men in a range from +1.3% to +45.8%). Among the individuals who increased the most their weight (over 20%), 2 women and 1 men were underweight (BMI < 18.5 kg/m2) when entering prison and 1 male inmate probably gain lean mass rather than fat mass because his HDL cholesterol was high (2.1 mM), his WC was only 96 cm and he was classified as highly physically active following the IPAQ test. Additionally, more men lost weight (33%) than women (21.2%) in a range from -1.6% to -15.1%. In both sexes, there was no association between weight gain and the duration of incarceration (Fig 1B).

Between prison entry and the time of the study the percentage of overweight participants increased from 24.2% (8/33) to 30.3% (10/33) in women and decreased from 16.7% (3/18) to 11% (2/18) in males while obesity increased from 18.2% (6/33) to 21.2% (7/33) in women and from 11.1% (2/18) to 16.7% (3/18) in men (Fig 1A).

A large gender difference was observed for WC: 69.7% (23/30) of female vs. 27.8% (3/18) of male inmates (p = 0.0004) being above thresholds considered for abdominal obesity (Fig 1C). Additionally, 33.0% (11/33) of women vs. 0% of men had metabolic syndrome (p = 0.005) (Fig 1D). Eight of these 11 women with metabolic syndrome are regular smokers (72.7%). An atherogenic index over 4.5 was present in 27.5% of inmates regardless of gender. Five of the women with the metabolic syndrome (45.5%) also had an atherogenic index above 4.5. Of note, among the 11 female subjects presenting a metabolic syndrome, only 1 woman was declared diabetic with a specific diet provided to her as well as statin medication.

#### Mood impairment, emotional vulnerability and eating behavior

Study participants scores for the psychometric tests are shown in Table 2.

**Table 2:** Study participants’ scores for the psychometric tests.

		Women N = 33	Men N = 18	P value
**Depressive symptoms (BDI)**				
	Median (IQR)	13.0 (12)	12.0 (8)	0.67
	Severe (≥16) %, (n)	24.2 (8)	35.3 (6)	0.51
**Anxiety symptoms (STAI-Y), Median (IQR)**				
	STAI-YA		53.0 (30)	50.0 (22)	0.67
	STAI-YB		51.0 (23)	50.0 (10.5)	0.92
**Coping (WCC), Median (IQR)**				
	self-blame	10.0 (6)	12.0 (5)	0.32
	escape avoidance	20.0 (6)	20.5 (4)	0.80
	seeking social support	13.0 (5)	14 (4)	0.66
	positive reappraisal	14.5 (5)	14.5 (3)	1.00
	planful problem solving	24.0 (8)	22.5 (6)	0.40
**Traumatic history (THQ): nb of events, Median (IQR)**				
	general disaster/trauma	4.0 (3)	5.5 (5)	0.11
	crime	1.0 (3)	1.5 (3)	0.72
	physical/sexual assaults	2.5 (4)	1.0 (2)	0.16
**Eating disorder (QEWP &DEBQ), Median (IQR)**				
	QEWP score	2.0 (9)	1.0 (4)	0.08
	BED (QEWP>8) %, (n)	27.3 (9)	11.1 (2)	0.29
	DEBQ_Restrained eating	2.3 (1.4)	2.0 (0.8)	0.60
	DEBQ _Emotional eating	1.9 (1.8)	1.9 (1.3)	0.37
	DEBQ_External eating	2.5 (0.7)	2.9 (1.2)	0.26

BDI = Beck Depression Inventory, STAI = Spielberger State-Trait Anxiety Inventory Y form, A (state) and B (trait), WCC = Way of Coping Checklist, THQ = Traumatic History Questionnaire, DEBQ = Dutch Eating Behavior Questionnaire, QEWP = Questionnaire on Eating and Weight Patterns, BED = Binge Eating Disorder.

Both women and men displayed high depressive and anxiety symptoms compared to norms but there was no statistical difference between genders for Beck, STAI-A & -B scores. Trait and state anxiety measures were highly correlated (STAI-A and STAI-B scores, r = 0.83, p<10^−6^) and both were also positively correlated to Beck scores (r = 0.68, p<10^−6^ for Beck-STAI-A and r = 0.77, p<10^−6^ for Beck-STAI-B). When classified according to severity score, 24.2% of female and 33.3% of male inmates had severe depressive symptoms (Beck score ≥16). Regarding coping strategies, equivalent scores were found between genders for each dimension. Interestingly, for both genders, scores were more elevated in the coping dimensions of planful problem solving and escape avoidance. For trauma history, there was no statistical difference between gender for total number of events of each trauma type, although the average number and frequency of each type of physical and sexual assaults experienced by imprisoned women were always higher than for imprisoned men (e.g. “unwanted intercourse/sex” 46.9% vs. 5.5%, “being beaten” 43.8% vs. 33%).

The QEWP test revealed a non-significant trend (p = 0.08) for higher eating disorder in women compared to men (2.0 vs. 1.0 for continuous score). Binge eating disorder (BED, QEWP score >8) was present in 27.3% of women and 11.0% of men, which is elevated compared to a nonpatient community sample where less than 5% reached this score of 8 [10]. In this study the gender difference was not statistically significant. No gender differences were detected for any of the three dimensions evaluated by the DEBQ although women tended to score higher than men for each dimension. QEWP score were positively correlated with depressive (r = 0.31, p<0.05) but not anxiety symptoms. QEWP scores were also positively correlated with DEBQ emotional and external eating scores (r = 0.29, p<0.05 for both) and close to significance for restrained eating (r = 0.26, p = 0.07).

#### Physical activity

**Fig 2:**
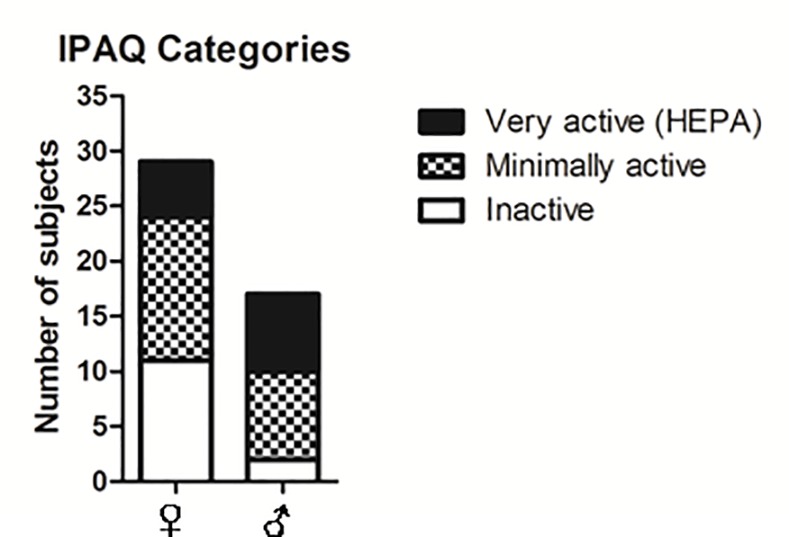
Participants distribution in IPAQ (International Physical Activity Questionnaire) Categories. HEPA = Health Enhancing Physical Activity.

A marked difference was found for physical activity between genders (Table 3). Higher scores in men were particularly significant for total MET scores (p = 0.007) and for time spent walking per day (p = 0.01). There was also a trend for number of days per week doing intense activity (p = 0.09). When physical activity was considered as categories (Fig 2), a trend for gender differences is also observed (p = 0.08): a higher proportion of women compared to men were inactive (37.9% vs. 11.8%) but fewer women were very active (17.2% vs. 41.2%). Among the 11 women with metabolic syndrome, 10 are classified as either inactive or minimally active (90.9%) after IPAQ test.

**Table 3:** Physical activity estimated by IPAQ (International Physical Activity Questionnaire) scores.

	Women	Men	P value
	n = 29	n = 17	
IPAQ scores Median (IQR)							
Total (MET/min/week)	1141.0 (2395)	2559.0 (3597)	p = 0.007
Number days/week of intense activity	0 (1)	0 (5)	p = 0.09
Number days/week of moderate activity	1.0 (2,5)	0 (4)	p = 0.45
Number days/week of walking	5.0 (4)	5.0 (4)	p = 0.85
Time min/day intense activity	0 (30)	0 (90)	p = 0.22
Time min/day moderate activity	15.0 (90)	0 (30)	p = 0.28
Time min/day walking	20.0 (50)	60.0 (75)	p = 0.01

MET = Metabolic Equivalent Task.

### Association of adiposity with mood, eating behavior and physical activity

As shown in Table 4, inmates with abdominal obesity (n = 26) were more often women (p≤0.003), and had a significantly lower total IPAQ MET score (IPAQ MET: p = 0.03) than the others (n = 25). Moreover, inmates with abdominal obesity displayed higher systolic blood pressure (SBP: p = 0.05), higher LDL cholesterol (LDL: p = 0.07), higher restrained eating (DEBQ restrained eating: p = 0.06) and higher QEWP score (p = 0.07) with borderline significance compared to inmates without abdominal obesity.

**Table 4:** Characteristics of inmates stratified according to risk waist circumference (WC), risk WC = 1 when WC≥ 80 cm for women and WC≥94 cm for men.

Abdominal adiposity	Total Population (N = 51)						
	WC = 0	N per category	N remaining	WC = 1	N per category	N remaining	P-value
	(N = 25)			(N = 26)			
Sex (% female)	40.0	10	25	88.5	23	26	**0.003**
Age, median (IQR)	40.0 (14)		25	41.0 (14)		26	0.87
Education (%)			25			26	0.73
*None/ Primary / Secondary*	84.0	21		76.9	20		
*Over*	16.0	4		23.1	6		
Family status (%)			25			26	0.86
*Married/ in couple*	44.0	11		42.3	11		
*Divorced/ separated*	28.0	7		34.6	9		
*Single*	28.0	7		23.1	6		
Number of children, Median (IQR)	1.0 (2)		25	3.0 (4)		26	0.09
Number of visits median (IQR)	2.0 (4)		25	1.5 (4)		26	0.81
First incarceration (%)	72.0	18	25	53.9	14	26	0.18
Sentenced (%)	44.0	11		65.4	17	26	0.13
Glycemia, median (IQR)	4.8 (0.4)		24	4.8 (0.6)		25	0.99
HbA1c, median (IQR)	5.5 (0.6)		24	5.6 (0.3)		25	0.74
HDL cholesterol, median (IQR)	1.2 (0.6)		24	1.4 (0.4)		25	0.38
LDL cholesterol, median (IQR)	3.3 (0.9)		23	3.9 (1.6)		25	**0.07**
Triglycerids, median (IQR)	1.3 (1.2)		24	1.2 (1)		25	0.80
SBP, median (IQR)	11.0 (2)		25	12.5 (3)		26	**0.04**
DBP, median (IQR)	7.0 (1)		25	8.0 (1)		26	0.32
Smoking (%)	52.0	13	25	73	19	26	0.33
BDI, median (IQR)	12.0 (10)		24	12.5 (12)		26	0.73
STAI-YA, median (IQR)	53.0 (25)		23	52.5 (23)		24	0.70
STAI-YB, median (IQR)	50.0 (11)		23	50.5 (18.5)		24	0.58
THQ, median (IQR)							
*Total physical/sexual assaults*,)	1.0 (3)		25	1.0 (3)		25	0.93
*Total crime*	1.0 (2)		25	2.0 (3)		25	0.38
*Total general disaster/trauma*,	4.0 (4)		25	4.0 (3)		25	0.67
Coping, median (IQR)							
*Self-blame*	10.0 (5)		21	11.0 (5)		21	0.82
*Escape avoidance*	20.0 (5)		21	20.0 (6)		21	0.86
*Seeking social support*	14.0 (4)		21	13.0 (4)		21	0.58
*Positive reappraisal*	15.0 (4)		21	14.0 (4)		21	0.51
*Planful problem solving*	22.0 (7)		21	23.0 (6)		21	0.52
DEBQ, median (IQR							
*External eating*,*)*	2.75 (1.1)		24	2.5 (0.8)		26	0.22
*Restrained eating*	1.95 (1.2)		24	2.4 (1.6)		26	**0.06**
*Emotional eating*	2.25 (1.3)		25	1.9 (1.8)		26	0.98
QEWP, median (IQR)	2.0 (5)		25	2.5 (9)		26	**0.07**
IPAQ (MET), median (IQR)	2559.0 (3653)		23	1182.0 (2049)		23	**0.03**
IPAQ category (%), n			23			23	0.26
*IPAQ inactive*	17.4	4		39.1	9		
*IPAQ minimally active*	52.2	12		39.1	9		
*IPAQ very active*	30.4	7		41.7	5		

HbA1c = glycated hemoglobin, SBP = systolic blood pressure, DBP = dystolic blood pressure, BDI = Beck depression Inventory, STAI = Spielberger State-Trait Anxiety Inventory Y form, A (state) and B (trait), THQ = traumatic history questionnaire, DEBQ = Dutch Eating Behavior Questionnaire, QEWP = Questionnaire on Eating and Weight Patterns, IPAQ = International Physical Activity Questionnaire.

Regarding determinants significantly associated with abdominal obesity in univariate analysis, we have performed multivariate analysis to identify those which were independently associated with abdominal obesity. In this analysis, IPAQ and QEWP were considered as main explanatory variables. After a backward elimination procedure, abdominal obesity was significantly associated with systolic blood pressure independently of IPAQ categories (Table 5). Indeed, a higher prevalence of abdominal obesity was observed with higher blood pressure (p = 0.03) and IPAQ inactive category (p = 0.04), while QEWP scores were not associated with abdominal obesity (p = 0.30) in the fully adjusted model.

**Table 5:** Association of abdominal obesity with physical activity (IPAQ category), eating disorder (QEWP score) and blood pressure (SBP).

Exact Odds Ratios				
Parameter	Estimate	95% Confidence Limits		Bilateral p-Value
**SBP**	1.605	1.032	2.697	0.0340
**QEWP**	1.094	0.929	1.308	0.2976
**IPAQ**	0.328	0.088	0.972	0.0429

Multivariate exact logistic regression.

## Discussion

This study shows that at entry into prison, women and men are frequently diagnosed as obese, as already reported by Herbert et al, 2012 [2] for high-income countries. At the time of study the prevalence of obesity had increased for both women and men. These data underline that the issue of obesity was already present in these women before entering prison and that incarceration worsens the prevalence of obesity, especially in men. Our data also show that participants underweight at prison entry gained weight following imprisonment pointing to a positive effect of incarceration on these subjects’ weight. A significant proportion of women lost weight (21.2%). Among those, 2 were obese and 2 others had a high WC (≥80), the rest lost little weight (2 or 3 kg) and none become underweight, again suggesting that incarceration improved the weight status of some subjects. Potential reasons may be a better balanced diet through the provided meals and limited snacking outside the meals for these subjects.

Regarding the scarce literature on this topic, the proportion of overweight or obese women in our study (51,2% with BMI>25) is in the lower range of what was found in the most recent surveys for female inmates in Spain (59.1%,[12]) and Australia (58%,[13]) and considerably lower than in USA (70%, [14]) but higher than in the study done in UK (40% after 1 month of incarceration [15]). A more recent longitudinal study from a statewide department of corrections in the east south central region of USA (2715 men and 217 women) found that prisoners’ BMI gain across a 7-year period was particularly high in women, change in BMI for female prisoners being on average 5.34 while only 0.67 in male prisoners [16]. The comparison with the present study is difficult because the duration of incarceration was much variable in this French cohort. However, the maximum of BMI change found was 0.45 in one of the woman, again arguing for much less weight gain than in USA. In any case, in our study and abroad, a large proportion of imprisoned women were overweight or obese before prison and imprisonment worsens their weight.

Besides BMI, WC was measured to estimate abdominal obesity because the latter is more deleterious for health than peripheral obesity. Women appeared to present considerably higher prevalence of abdominal obesity than men but close to French norms (i.e. 67.6% for women and 52.3% for men using the same thresholds). In the recent Spanish survey [12], abdominal obesity was also found considerably higher in female inmates compared to males: 54.4% of women had a risk waist circumference against 15.2% of men, using thresholds from US National Cholesterol Education Program (NCEP). With these norms, 48.4% of the imprisoned women and 5.5% of men of this study’s sample are above the thresholds for cardiovascular risk, while in the French population there are 43% of women and 27% of men above these thresholds [3]. These observations were reinforced by analyzing the presence of metabolic syndrome which, again, is elevated in imprisoned women but not in men of this study’s sample. Conversely, cardiovascular risk estimated by the atherogenic index was equivalent in male and female inmates of this study’s sample while in the Spanish survey it was present in 44.4% of men and 23.9% of women. The present study probably underestimates obesity and its complications in men but confirms that cardiovascular- and diabetes-risk associated obesity is particularly high in imprisoned women. Because these data were not available from subjects at prison admittance we cannot conclude whether abdominal obesity and metabolic syndrome have appeared during incarceration or not.

No gender differences were observed for depressive and anxiety symptoms and no relationships were found with either obesity or metabolic parameters. For both sexes, these scores are high compared to the norms from healthy populations. Similarly, neither gender differences nor correlation with obesity were found for each coping scores or for the number of traumatic events experienced by female vs. male subjects or inmates with abdominal obesity vs. non-obese subjects. Absence of gender differences for emotional traits was unexpected since the prevalence of mental illness was already reported as higher in female than in male prisoners for depression, anxiety and post-traumatic stress disorder [17,18,19]. This apparent discrepancy can be explained by various factors: (i) the size of the enrolled male sample is small and a bias towards highly depressive and anxious men may have occurred (ii) different psychometric tools were used to assess depressive and anxiety symptoms across studies (iii) there is no published data from French prisons to date. The lack of association between mood and obesity may stem from a ceiling effect with all inmates having such high depressive and anxiety symptoms, poor coping and heavy trauma history that obesity cannot be discriminative.

Eating behavior evaluated by the QEWP questionnaire revealed a tendency for higher eating disorder in imprisoned women vs. men, both having higher scores of binge eating disorder than the norms. Inmates with abdominal obesity had also a clear tendency for higher scores of eating disorder than those without abdominal obesity although in multivariate analysis QEWP scores alone did not explain abdominal obesity.

There was no gender differences for eating behavior evaluated through the DEBQ although women scored higher for emotional and restrained eating and less for external eating. Interestingly, inmates with abdominal obesity showed a tendency for higher restrained eating.

The positive correlations found between BED, depressive symptoms and emotional eating is often found in obese patients with BED. Indeed, negative emotions, tension and instability are antecedents of binge eating which is for the subjects intended to serve the function of reducing negative emotions [20]. Restrained eating is also often encountered in obese persons that overeat after a period of slimming when the resolution to diet is abandoned [11].

Finally, physical activity was found to be a major factor explaining both gender differences and abdominal obesity. IPAQ total Met scores medians are over 2 times lower in women than men and also over 2 times lower in inmates with abdominal obesity compared to participants without abdominal obesity. The factor IPAQ category remains significant as an explanatory factor of abdominal obesity in multivariate analysis. The data collected suggest that most women are inactive or minimally active while most men are either minimally active or very active. Although the variability is high within genders, women spend less time walking and do less intense physical activities. The opportunity for prisoners to exercise takes place either during the time they are allowed to go for a walk in the prison yard, twice a day for 1 hour, or during organized sport programs occurring once or twice a week for 2 hours and on a voluntary basis. The present findings suggest that women would do less walking during their time out in the prison yard and that they would not engage as much as men into sport programs to do intense physical activities. Few studies have looked at physical activity in prison and the relationship with overweight or obesity. In their systematic survey, Herbert et al [2] reported on six studies (4 in Australia and 2 in UK) that adequate physical activity (over 150 min per week) is achieved in Australian but not in UK prisons for both male and female inmates. In the Spanish survey, sedentary life (<30 min physical activity per day) was found linked to diabetes prevalence in prison [12].

### Limitations

We acknowledge several limitations to our study. First, the size of the population is small, particularly for men. We were unable to recruit a representative male population from the same institution or even an equivalent absolute number, mainly because male inmates were not interested in the study. Nevertheless, we decided to present the gender differences observed as an indication for further studies. Second, our findings are based on data from a single French prison located in the south western part of France. Variability in several of the assessments done in this study probably exists between correctional institutions. Regarding overweight and obesity, this part of France is less affected than the general French population [3] which suggest that the present observations will presumably be more alarming in other institutions. A third limitation is that our data are based on self-report and, as such, raises the possibility of under-reporting because of social desirability concerns. Again, if a social desirability bias was operating, the present results would be all the more alarming. Fourth, we have not examined the energy intake of the inmates. Meals are provided by the prison and the content is highly controlled to offer a balanced nutrition but there are no differentiations between men and women who are both served a diet of ~2400 kcal/d. Inmates eat in their cell, on their own for most women. In addition, inmates can purchase food at the prison store. Thus, the energy intake is variable between prisoners but probably above needs for most of them.

### Conclusion

This study provides new data highlighting obesity as a health problem in women incarcerated in a French prison. The population studied is small but it has the advantage that multiple confounding factors were considered. Obesity is an issue before incarceration in women and is aggravated after, although moderately in this sample compared to other high-income countries. In addition to obesity, metabolic syndrome, binge eating and a high level of anxio-depressive symptoms are present. Since smoking is frequent and the practice of physical activity low, these women are at high risk of cardiovascular disease. Physical activity seems to be the major difference with male prisoners. Therefore, increased physical exercise, if well suited for obese women, combined with a healthier nutrition encouraged by nutritional education, appear as factors on which interventions should be focused.
